# Design, Synthesis and Antifungal Activity of Novel Benzofuran-Triazole Hybrids

**DOI:** 10.3390/molecules21060732

**Published:** 2016-06-07

**Authors:** Zhen Liang, Hang Xu, Ye Tian, Mengbi Guo, Xin Su, Chun Guo

**Affiliations:** 1 Key Laboratory of Structure-Based Drugs Design & Discovery of Ministry of Education, Shenyang Pharmaceutical University, Shenyang 110016, China; llzz_666@126.com (Z.L.); xuhanglg2@126.com (H.X.); tianye870918@126.com (Y.T.); guomengbi@hotmail.com (M.G.); 2 School of Life Sciences and Biopharmaceutics, Shenyang Pharmaceutical University, Shenyang 110016, China; suxin1966@163.com

**Keywords:** antifungal activity, *N*-myristoyltransferase, benzofuran, 1,2,3-triazole, click chemistry, molecule hybrid

## Abstract

A series of novel benzofuran-triazole hybrids was designed and synthesized by click chemistry, and their structures were characterized by HRMS, FTIR and NMR. The *in vitro* antifungal activity of target compounds was evaluated using the microdilution broth method against five strains of pathogenic fungi. The result indicated that the target compounds exhibited moderate to satisfactory activity. Furthermore, molecular docking was performed to investigate the binding affinities and interaction modes between the target compound and *N*-myristoyltransferase. Based on the results, preliminary structure activity relationships (SARs) were summarized to serve as a foundation for further investigation.

## 1. Introduction

Fungal infections have posed a continuous and serious threat to human health and life during the past two decades, especially among hosts, such as patients undergoing anticancer chemotherapy or organ transplants, and patients with AIDS [1,2]. Clinically, available antifungal drugs have several drawbacks, such as drug-related toxicity, non-optimal pharmacokinetics, and the emergence of drug resistance [3,4]. Therefore, the development of new antifungal drugs with novel modes of action is required.

**Figure 1:**
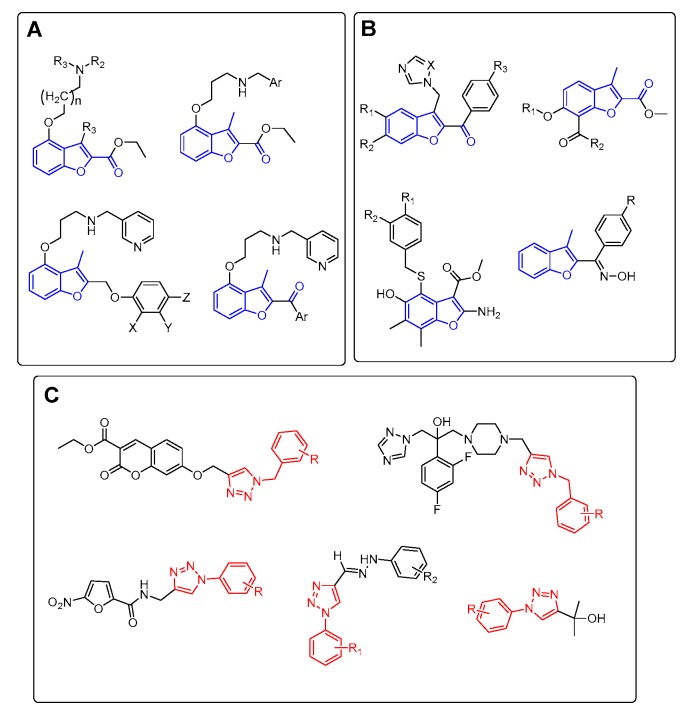
The representative structures of antifungal agents. (**A**) Representative structures of the benzofuran NMT inhibitors; (**B**) Representative benzofuran derivatives with antifungal activity; (**C**) Representative 1,2,3-triazole derivatives with antifungal activity.

*N*-Myristoyltransferase (NMT) is a monomeric enzyme that catalyzes the transfer of the myristoyl group of myristoyl-CoA to the *N*-terminal glycine of various eukaryotic cellular proteins [5,6] and it was proven to be essential for the viability of pathogenic fungi, such as *C. albicans* [7] and *C. neoformans* [8]. Although NMT is also distributed in mammalian cells, there are clear differences in the peptide-substrate specificity between human and fungal NMT [9], which could be exploited to avoid adverse events caused by inhibiting human NMT. Therefore, NMT would be a promising target for the development of novel fungicidal drugs. Up to now, various types of NMT inhibitors such as peptidomimetic [10,11,12], myristic acid analogues [13] and different kinds of heterocycles [14,15,16] have been reported. Among them, benzofuran inhibitors showed high selectivity and powerful antifungal activity [16,17,18,19] (Figure 1). Furthermore, the benzofuran core itself possessed a definite antifungal activity and numbers of benzofuran derivatives were reported without indicating the targets [20,21,22,23] (Figure 1).

In recent years, the 1,2,3-triazole scaffold became a highlight fragment with the emergence of click chemistry and the 1,2,3-triazole–containing compounds were reported to possess a variety of biological activities [24,25,26,27,28], especially as antifungal agents [29,30,31]. Furthermore, the hybridizations of the 1,2,3-triazole moiety with other antifungal agents were reported. Coumarin derivatives incorporating the 1,2,3-triazole moiety showed antifungal activity [32]. Hybrids of fluconazole with 1,2,3-triazole were proved to possess satisfactory activity [33].

Encouraged by the results above, we attempted to design and synthesize a series of benzofuran-triazole hybrids to evaluate the *in vitro* antifungal activity.

## 2. Results and Discussion

### 2.1. Chemistry

**Scheme 1:**
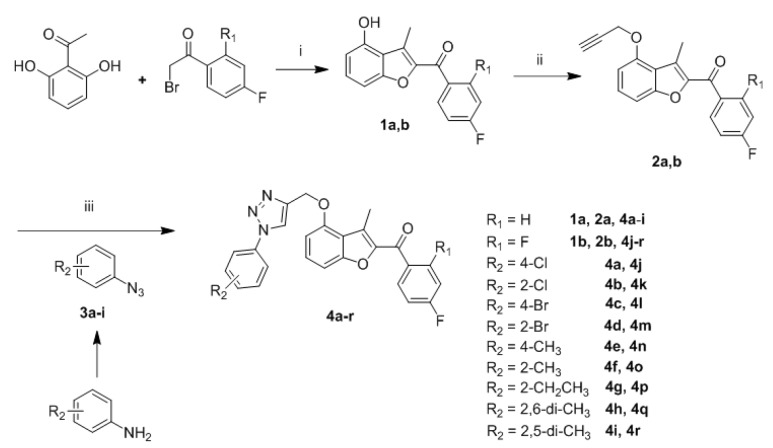
Synthesis of target compounds **4a**–**r**. Reagents and conditions: (i) K_2_CO_3_/CH_3_CN/reflux 5–6 h; (ii) propargyl bromide/K_2_CO_3_/r.t. 3–5 h; (iii) CuSO_4_∙5H_2_O/sodium ascorbate/DMF/r.t. 2–4 h.

The synthetic route to target compounds was outlined in Scheme 1. The reaction of 2′,6′-dihydroxyacetophenone with the corresponding 2-bromoacetophenone in a modified Rap-Stormer reaction condition [22] gave the benzofuran scaffold (**1**). Alkylation of the hydroxyl group of **1a**,**b** with propargyl bromide gave terminal alkyne derivatives (**2a**,**b**). The aromatic azides (**3a**–**i**) were prepared from the corresponding anilines following the Sandmeyer conditions [27,31]. Finally, employing click chemistry, compounds **2a**,**b** were cyclized with **3a**–**i**, respectively, to give target compounds **4a**–**r** in good yields.

### 2.2. Antifungal Activity

The *in vitro* antifungal activity of the target compounds was measured by means of the minimal inhibitory concentrations (MICs) with fluconazole as the control drug. The results are summarized in Table 1. Against fluconazole-resistant *Trichophyton rubrum*, many target compounds (**4e**,**f**, **4h** and **4b**–**r**) showed better activity than fluconazole (128 μg·mL^−1^) in the range of 32 to 64 μg·mL^−1^, and some compounds (**4b**, **4d**, **4g**, and **4i**–**l**) showed equivalent activity to fluconazol. Except compounds **4a** and **4c**, most of the compounds showed antifungal activity against *Cryptococcus neoformans* in concentrations ranging from 32 to 128 μg·mL^−1^. The target compounds (**4f**, **4h**, **4m**, **4p** and **4r**) showed antifungal activity against *Candida zeylanoides* at the concentration of 64 μg·mL^−1^. Some compounds showed weak activity merely against *Candida albicans* (**4f**, **4h**, **4m**, **4o** and **4r**) and *Rhodotorula rubra* (**4d**, **4f**, **4h**, **4o** and **4q**,**r**) at the concentration of 128 μg·mL^−1^.

Observing the antifungal assay results, it can be noticed that the derivatives with a di-fluorine-substituted phenyl ring at the benzofuran C-2 side chain (ring A) are more effective than the mono-fluorine ones (e.g., **4f** *vs.* **4o**). Meanwhile, the substituted groups on the phenyl ring linked to the triazole (ring B) also had an impact on the activity. The alkyl-substituted compounds are more potent than the halogenated derivatives (e.g., **4b** *vs.* **4f**) and the ortho-substituted derivatives are more potent than the para isomers (e.g., **4e** *vs.* **4f**, **4j** *vs.* **4k**). The preliminary structure activity relationships (SARs) were supported by the outstanding bioactivities of **4o** and **4r** among all the target compounds.

### 2.3. Molecule Docking

**Figure 2:**
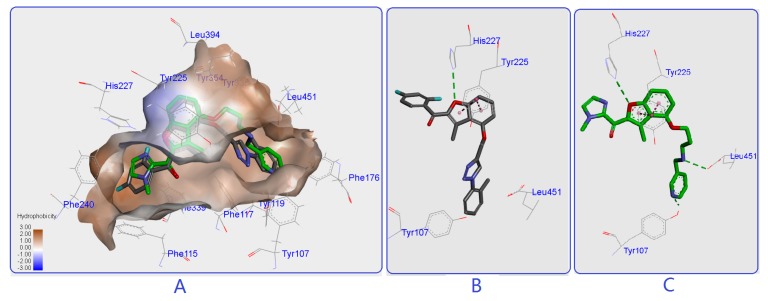
Molecule docking results.(**A**) The location of docking ligands; (**B**) Hydrogen bonds between **4o** and receptor; (**C**) Hydrogen bonds between co-crystal ligand and receptor.

In an attempt to investigate the action modes of the target compounds, **4o** was docked into the crystal structure of NMT from *C. albicans* (CaNMT, PDB ID: 1IYL) using Discovery Studio 3.0. The docking results are illustrated in Figure 2. The benzofuran ring was located at the center of the active site, surrounding some hydrophobic residues, such as Tyr225, Tyr354 and Leu394, and forming a pi-pi interaction with Tyr225. The di-fluorine phenyl fragment formed a hydrophobic interaction with Phe115, Phe240 and Phe339. The phenyl triazole side chain stretched into the hydrophobic pocket constituted by Phe117, Tyr119 and Phe176. The oxygen atom of the benzofuran ring formed a hydrogen bond with His227. The interaction mode between **4o** and the receptor is similar to the co-crystal ligand. On the other hand, no hydrogen bond was observed between triazole and Leu451, which is formed between the C-4 secondary amine of the co-crystal ligand. This hydrogen bond is important to the antifungal activity [34]. The docking result is supported by the weak antifungal activity against *C. albicans* of the target compounds.

## 3. Experimental Section

### 3.1. Chemistry

The ^1^H- and ^13^C-NMR spectra were recorded respectively on a Bruker AV-600 spectrometer and a Bruker AV-400 spectrometer. Chemical shifts were reported in parts per million (ppm, δ) downfield from TMS as an internal standard. High-resolution mass spectra (HRMS) were measured with an Agilent Accurate-Mass Q-TOF 6530 in ESI mode (Agilent, Santa Clara, CA, USA). FTIR spectra were recorded on a Bruker IFS 55 spectrometer (Bruker Co., Karlsruhe, Germany). Melting points (m.p.) were determined on an X-4 microscope melting point apparatus (Beijing Tech instrument Co., Ltd., Beijing, China) without calibration.

#### 3.1.1. General Procedure for the Synthesis of Compounds **1a**,**b**

The 2′,6′-Dihydroxyacetophenone (7.60 g, 50 mmol), potassium carbonate (8.30 g, 60 mol) and substituted 4-bromoacetylbenzene (50 mmol) were refluxed in 80 mL acetonitrile for six hours. After cooled to room temperature, the reaction mixture was poured into water. The crude products were filtrated and crystallized from ethanol.

*2-(4-Fluorobenzoyl)-3-methyl-4-hydroxybenzofuran* (**1a**): Yellow solid; yield 58%. ^1^H-NMR (600 MHz, DMSO-*d*_6_) δ 10.51 (s, 1H), 8.04 (dd, *J* = 8.5, 5.6 Hz, 2H), 7.39 (t, *J* = 8.7 Hz, 2H), 7.30 (t, *J* = 8.1 Hz, 1H), 7.03 (d, *J* = 7.9 Hz, 1H), 6.68 (d, *J* = 7.9 Hz, 1H), 2.70 (s, 3H). ^13^C-NMR (151 MHz, DMSO) δ 183.72, 165.78, 164.12, 155.81, 155.22, 146.39, 134.61, 132.58, 132.51, 130.15, 128.01, 117.89, 115.99, 115.85, 108.47, 103.14, 12.01. IR (KBr, cm^−1^): 3423, 2924, 1623, 1604, 1385, 1269, 1054. ESI–HRMS (*m*/*z*) found: 269.0619 (calcd. for C_16_H_10_FO_3_ [M − H]^−^: 269.0619).

*2-(2,4-Difluorobenzoyl)-3-methyl-4-hydroxybenzofuran* (**1b**): Yellow solid; yield 62%. ^1^H-NMR (600 MHz, DMSO-*d*_6_) δ 10.57 (s, 1H), 7.78–7.71 (m, 1H), 7.46–7.43 (m, 1H), 7.33–7.23 (m, 2H), 6.97 (d, *J* = 7.9 Hz, 1H), 6.67 (d, *J* = 7.9 Hz, 1H), 2.67 (s, 3H). ^13^C-NMR (151 MHz, DMSO) δ 181.82, 165.34, 163.67, 156.06, 155.45, 146.27, 132.47, 130.71, 128.14, 118.05, 112.61, 112.45, 108.47, 103.07, 11.67. IR (KBr, cm^−1^): 3417, 2925, 1614, 1385, 1269, 1054. ESI-HRMS (*m*/*z*) found: 287.0531 (calcd. for C_16_H_9_F_2_O_3_ [M − H]^−^: 287.0525).

#### 3.1.2. General Procedure for the Synthesis of Compounds **2a**,**b**

To a solution of compound **1a** or **1b** (10.0 mmol) in DMF (20 mL), propargyl bromide (1.30 g, 10.9 mmol) and potassium carbonate (1.70 g, 12.3 mmol) were added. The reaction mixture was stirred at room temperature for 5 h, and was then diluted with ethyl acetate (80 mL) and washed with water (2 × 100 mL). The organic layer was dried over anhydrous Na_2_SO_4_ and concentrated under reduced pressure. The crude products were used without purification.

*2-(4-Fluorobenzoyl)-3-methyl-4-(propyn-3-yloxy)benzofuran* (**2a**): Yellow solid; yield 62%. ^1^H-NMR (600 MHz, DMSO-*d*_6_) δ 8.07–8.04 (m, 2H), 7.48 (t, *J* = 8.2 Hz, 1H), 7.43–7.36 (m, 2H), 7.26 (d, *J* = 8.3 Hz, 1H), 6.93 (d, *J* = 8.1 Hz, 1H), 4.98 (d, *J* = 2.4 Hz, 2H), 3.66 (t, *J* = 2.4 Hz, 1H), 2.69 (s, 3H). ^13^C-NMR (151 MHz, DMSO) δ 183.80, 165.88, 164.21, 155.27, 154.47, 146.86, 134.46, 132.67, 132.61, 130.01, 127.15, 118.69, 116.04, 115.90, 106.11, 105.87, 79.16, 56.62, 11.99. IR (KBr, cm^−1^): 3298, 2925, 2115, 1642, 1601, 1501, 1271, 1257, 1236, 1089. ESI-HRMS (*m*/*z*) found: 331.0741 (calcd. for C_1__9_H_13_FO_3_Na [M + Na]^+^: 331.0741).

*2-(2,4-Difluorobenzoyl)-3-methyl-4-(propyn-3-yloxy)benzofuran* (**2b**): Yellow solid; yield 55%. ^1^H-NMR (600 MHz, DMSO-*d*_6_) δ 7.77 (td, *J* = 8.3, 6.5 Hz, 1H), 7.50–7.44 (m, 2H), 7.28 (td, *J* = 8.5, 2.5 Hz, 1H), 7.20 (d, *J* = 8.4 Hz, 1H), 6.92 (d, *J* = 8.1 Hz, 1H), 4.98 (d, *J* = 2.4 Hz, 2H), 3.66 (t, *J* = 2.4 Hz, 1H), 2.68 (s, 3H). ^13^C-NMR (151 MHz, DMSO) δ 181.95, 165.39, 163.80, 155.50, 154.65, 146.70, 132.62, 130.57, 127.26, 124.06, 118.80, 112.66, 112.50, 106.15, 105.79, 79.21, 56.66, 11.67. IR (KBr, cm^−1^): 3239, 2116, 1641, 1612, 1499, 1086. ESI-HRMS (*m*/*z*) found: 349.0646 (calcd. for C_1__9_H_12_F_2_O_3_Na [M + Na]^+^: 349.0647).

#### 3.1.3. General Procedure for the Synthesis of Compounds **4a**–**r**

Compound **2a** (305 mg, 1 mmol), CuSO_4_·5H_2_O (50 mg, 0.2 mmol) and 4-chloroazidobenzene (135 mg, 1 mmol) were dissolved in 10 mL DMF, and sodium ascorbate (99 mg, 0.5 mmol) was added. The reaction mixture was stirred at room temperature for 3 h, then was poured into water. The crude product was filtered, which was purified by silica gel column chromatography (PET/EtOAc = 2:1, *v*/*v*) to give target compound **4a** as yellow solid; yield 72%; m.p.: 168–170 °C. ^1^H-NMR (600 MHz, DMSO-*d*_6_) δ 9.02 (s, 1H), 8.04 (dd, *J* = 8.4, 5.7 Hz, 2H), 7.96 (d, *J* = 8.7 Hz, 2H), 7.66 (d, *J* = 8.7 Hz, 2H), 7.48 (t, *J* = 8.2 Hz, 1H), 7.38 (t, *J* = 8.7 Hz, 2H), 7.25 (d, *J* = 8.4 Hz, 1H), 7.09 (d, *J* = 8.1 Hz, 1H), 5.41 (s, 2H), 2.65 (s, 3H); ^13^C-NMR (101 MHz, DMSO) δ 183.85, 155.41, 155.32, 146.88, 144.22, 135.77, 133.55, 132.76, 132.66, 130.35, 130.32, 127.51, 123.33, 122.31, 118.74, 116.17, 115.95, 106.08, 105.78, 62.24, 12.13; IR (KBr, cm^−1^): 2925, 1635, 1599, 1553, 1502, 1271, 1252, 1087. ESI–HRMS (*m*/*z*) found: 484.0841 (calcd. for C_25_H_17_ClFN_3_O_3_Na [M + Na]^+^: 484.0835).

The compounds **4b**–**r** were synthesized using the same operation procedure of compound **4a**.

*Compound* **4b**: Yellow solid; yield 79%; m.p.: 169–170 °C. ^1^H-NMR (600 MHz, DMSO-*d*_6_) δ 8.78 (s, 1H), 8.07–8.02 (m, 2H), 7.78 (dd, *J* = 8.0, 1.4 Hz, 1H), 7.73 (dd, *J* = 7.8, 1.7 Hz, 1H), 7.64 (td, *J* = 7.8, 1.7 Hz, 1H), 7.59 (td, *J* = 7.7, 1.4 Hz, 1H), 7.50 (t, *J* = 8.2 Hz, 1H), 7.40 (t, *J* = 8.8 Hz, 2H), 7.26 (d, *J* = 8.4 Hz, 1H), 7.12 (d, *J* = 8.1 Hz, 1H), 5.44 (s, 2H), 2.65 (s, 3H). ^13^C-NMR (101 MHz, DMSO) δ 183.88, 155.41, 146.90, 143.10, 134.90, 134.59, 132.76, 132.67, 132.22, 131.04, 130.33, 129.00, 128.90, 127.50, 127.28, 118.78, 116.18, 115.96, 106.24, 105.79, 62.37, 12.06. IR (KBr, cm^−1^): 2923, 1638, 1598, 1556, 1500, 1272, 121232, 1091. ESI-HRMS (*m*/*z*) found: 484.0844 (calcd. for C_25_H_17_ClFN_3_O_3_Na [M + Na]^+^: 484.0835).

*Compound* **4c**: Yellow solid; yield 76%; m.p.: 172–174 °C. ^1^H-NMR (600 MHz, DMSO-*d*_6_) δ 9.04 (s, 1H), 8.04 (dd, *J* = 8.5, 5.6 Hz, 2H), 7.90 (d, *J* = 8.8 Hz, 2H), 7.80 (d, *J* = 8.8 Hz, 2H), 7.49 (t, *J* = 8.2 Hz, 1H), 7.39 (t, *J* = 8.8 Hz, 2H), 7.25 (d, *J* = 8.4 Hz, 1H), 7.10 (d, *J* = 8.0 Hz, 1H), 5.42 (s, 2H), 2.65 (s, 3H). ^13^C-NMR (101 MHz, DMSO) *δ* 183.87, 155.32, 146.88, 144.23, 136.18, 133.28, 132.76, 132.67, 130.33, 127.51, 123.31, 122.55, 121.93, 118.74, 116.17, 115.96, 106.09, 105.79, 62.24, 40.61, 12.14. IR (KBr, cm^−1^): 2924, 1633, 1600, 1550, 1498, 1270, 1251, 1087. ESI-HRMS (*m*/*z*) found: 506.0515 (calcd. for C_25_H_18_BrFN_3_O_3_ [M + H]^+^: 506.0516).

*Compound* **4d**: Yellow solid; yield 81%; m.p.: 166–169 °C. ^1^H-NMR (600 MHz, DMSO-*d*_6_) δ 8.75 (s, 1H), 8.05 (dd, *J* = 8.6, 5.6 Hz, 2H), 7.93–7.90 (m, 1H), 7.68 (dd, *J* = 7.8, 1.3 Hz, 1H), 7.64–7.61 (m, 1H), 7.58–7.54 (m, 1H), 7.50 (t, *J* = 8.2 Hz, 1H), 7.40 (t, *J* = 8.8 Hz, 2H), 7.26 (d, *J* = 8.3 Hz, 1H), 7.12 (d, *J* = 8.1 Hz, 1H), 5.44 (s, 2H), 2.66 (s, 3H). ^13^C-NMR (101 MHz, DMSO) δ 183.87, 155.40, 146.89, 143.02, 136.58, 134.58, 134.11, 132.76, 132.67, 132.52, 130.33, 129.46, 129.20, 127.53, 127.25, 119.34, 118.78, 116.17, 115.95, 106.23, 105.77, 62.40, 12.09. IR (KBr, cm^−1^): 2923, 1638, 1614, 1598, 1555, 1500, 1271, 1255, 1090. ESI–HRMS (*m*/*z*) found: 506.0520 (calcd. for C_25_H_18_BrFN_3_O_3_ [M + H]^+^: 506.0516).

*Compound* **4e**: Yellow solid; yield 77%; m.p.: 160–161 °C. ^1^H-NMR (600 MHz, DMSO-*d*_6_) δ 8.96 (s, 1H), 8.04 (dd, *J* = 8.7, 5.6 Hz, 2H), 7.80 (d, *J* = 8.4 Hz, 2H), 7.49 (t, *J* = 8.2 Hz, 1H), 7.39 (m, *J* = 8.8, 4.7 Hz, 4H), 7.25 (d, *J* = 8.4 Hz, 1H), 7.10 (d, *J* = 8.1 Hz, 1H), 5.41 (s, 2H), 2.66 (s, 3H), 2.37 (s, 3H). ^13^C-NMR (101 MHz, DMSO) δ 183.85, 155.36, 146.87, 143.91, 138.90, 134.74, 134.55, 132.75, 132.66, 130.71, 130.32, 127.53, 123.12, 120.47, 118.73, 116.16, 115.94, 106.07, 105.74, 62.30, 21.05, 12.13. IR (KBr, cm^−1^): 2925, 1637, 1599, 1553, 1499, 1271, 1254, 1088. ESI-HRMS (*m*/*z*) found: 464.1393 (calcd. for C_26_H_20_FN_3_O_3_Na [M + Na]^+^: 464.1386).

*Compound* **4f**: Yellow solid; yield 80%; m.p.: 154–156 °C. ^1^H-NMR (600 MHz, DMSO-*d*_6_) δ 8.69 (s, 1H), 8.10–7.99 (m, 2H), 7.51–7.45 (m, 4H), 7.40 (q, *J* = 8.7 Hz, 3H), 7.26 (d, *J* = 8.5 Hz, 1H), 7.12 (d, *J* = 8.1 Hz, 1H), 5.43 (s, 2H), 2.65 (s, 3H), 2.13 (s, 3H). ^13^C-NMR (101 MHz, DMSO) δ 183.87, 166.37, 163.87, 155.41, 146.88, 143.08, 136.66, 134.55, 133.54, 132.76, 132.67, 131.84, 130.34, 127.50, 126.66, 126.52, 118.77, 116.17, 115.95, 106.18, 105.75, 62.50, 17.78, 12.04. IR (KBr, cm^−1^): 2925, 1639, 1624, 1599, 1544, 1501, 1271, 1250, 1234, 1089. ESI-HRMS (*m*/*z*) found: 464.1394 (calcd. for C_26_H_20_FN_3_O_3_Na [M + Na]^+^: 464.1386).

*Compound* **4g**: Yellow solid; yield 78%; m.p.: 155–158 °C. ^1^H-NMR (600 MHz, DMSO-*d*_6_) δ 8.67 (s, 1H), 8.04 (dd, *J* = 8.8, 5.6 Hz 2H), 7.54 (m, 1H), 7.51–7.47 (m, 2H), 7.42 (dd, *J* = 4.3, 1.5 Hz, 2H), 7.41–7.35 (m, 2H), 7.26 (d, *J* = 8.3 Hz, 1H), 7.11 (d, *J* = 8.1 Hz, 1H), 5.43 (s, 2H), 2.64 (s, 3H), 2.41 (q, *J* = 7.6 Hz, 2H), 0.96 (t, *J* = 7.6 Hz, 3H). ^13^C-NMR (101 MHz, DMSO) δ 183.86, 166.37, 163.88, 155.40, 146.90, 143.11, 139.81, 136.13, 134.58, 132.76, 132.66, 130.75, 130.38, 130.31, 127.49, 126.96, 118.80, 116.17, 115.95, 106.24, 105.74, 62.50, 24.27, 15.17, 11.99. IR (KBr, cm^−1^): 2925, 1626, 1598, 1541, 1501, 1269, 1249, 1088. ESI-HRMS (*m*/*z*) found: 456.1719 (calcd. for C_27_H_23_FN_3_O_3_ [M + H]^+^: 456.1723).

*Compound* **4h**: Yellow solid; yield 71%; m.p.: 156–159 °C. ^1^H-NMR (600 MHz, DMSO-*d*_6_) δ 8.58 (s, 1H), 8.04 (dd, *J* = 8.7, 5.6 Hz, 2H), 7.49 (t, *J* = 8.2 Hz, 1H), 7.45–7.34 (m, 3H), 7.29 (d, *J* = 7.6 Hz, 2H), 7.25 (d, *J* = 8.4 Hz, 1H), 7.09 (d, *J* = 8.0 Hz, 1H), 5.44 (s, 2H), 2.62 (s, 3H), 1.91 (s, 6H). ^13^C-NMR (101 MHz, DMSO) δ 183.86, 155.39, 146.90, 143.12, 136.22, 135.30, 134.57, 132.76, 132.67, 130.48, 130.30, 128.87, 127.46, 126.98, 118.80, 116.17, 115.95, 106.28, 105.74, 62.65, 17.24, 11.88. IR (KBr, cm^−1^): 2924, 1640, 1599, 1551, 1501, 1271, 1255, 1101, 1042. ESI-HRMS (*m*/*z*) found: 456.1716 (calcd. for C_27_H_23_FN_3_O_3_ [M + H]^+^: 456.1723).

*Compound* **4i**: Yellow solid; yield 70%; m.p.: 157–158 °C. ^1^H-NMR (600 MHz, DMSO-*d*_6_) δ 8.63 (s, 1H), 8.04 (dd, *J* = 8.7, 5.6 Hz, 2H), 7.49 (t, *J* = 8.2 Hz, 1H), 7.43–7.36 (m, 2H), 7.33 (d, *J* = 8.0 Hz, 1H), 7.28 (d, *J* = 1.8 Hz, 1H), 7.26 (d, *J* = 8.4 Hz, 1H), 7.20 (dd, *J* = 8.0, 1.9 Hz, 1H), 7.11 (d, *J* = 8.1 Hz, 1H), 5.42 (s, 2H), 2.65 (s, 3H), 2.35 (s, 3H), 2.08 (s, 3H). ^13^C-NMR (101 MHz, DMSO) δ 183.85, 163.87, 155.42, 146.88, 142.99, 140.02, 134.57, 134.29, 133.19, 132.76, 132.66, 132.23, 130.32, 127.89, 127.51, 126.66, 126.30, 118.77, 116.17, 115.95, 106.17, 105.73, 62.50, 21.10, 17.68, 12.03. IR (KBr, cm^−1^): 2925, 1639, 1600, 1553, 1502, 1270, 1255, 1101. ESI-HRMS (*m*/*z*) found: 456.1713 (calcd. for C_27_H_23_FN_3_O_3_ [M + H]^+^: 456.1723).

*Compound* **4j**: Yellow solid; yield 82%; m.p.: 168–171 °C. ^1^H-NMR (600 MHz, DMSO-*d*_6_) δ 9.04 (s, 1H), 7.99–7.94 (m, 2H), 7.75 (td, *J* = 8.4, 6.5 Hz, 1H), 7.69–7.64 (m, 2H), 7.49 (t, *J* = 8.2 Hz, 1H), 7.45 (td, *J* = 10.3, 2.3 Hz, 1H), 7.27 (td, *J* = 8.4, 2.2 Hz, 1H), 7.20 (d, *J* = 8.4 Hz, 1H), 7.09 (d, *J* = 8.1 Hz, 1H), 5.42 (s, 2H), 2.64 (s, 3H). ^13^C-NMR (101 MHz, DMSO) δ 182.80, 156.45, 15631, 147.55, 144.98, 136.58, 134.35, 133.46, 131.68, 131.16, 128.44, 124.15, 123.12, 119.66, 113.39, 106.91, 106.50, 106.35, 106.09, 105.83, 63.08, 12.62. IR (KBr, cm^−1^): 2924, 1635, 1600, 1551, 1502, 1271, 1252, 1088. ESI-HRMS (*m*/*z*) found: 480.0917 (calcd. for C_25_H_17_ClF_2_N_3_O_3_ [M + H]^+^: 480.0927).

*Compound* **4k**: Yellow solid; yield 78%; m.p.: 166–168 °C. ^1^H-NMR (600 MHz, DMSO-*d*_6_) δ 8.78 (s, 1H), 7.80–7.75 (m, 2H), 7.73 (dd, *J* = 7.8, 1.7 Hz, 1H), 7.64 (td, *J* = 7.8, 1.6 Hz, 1H), 7.59 (t, *J* = 7.6 Hz, 1H), 7.50 (t, *J* = 8.2 Hz, 1H), 7.46 (td, *J* = 10.3, 2.2 Hz,1H), 7.27 (td, *J* = 8.5, 2.2 Hz,1H), 7.21 (d, *J* = 8.4 Hz, 1H), 7.12 (d, *J* = 8.1 Hz, 1H), 5.45 (s, 2H), 2.63 (s, 3H). ^13^C-NMR (101 MHz, DMSO) δ 181.99, 155.64, 155.56, 146.75, 143.06, 134.89, 132.22, 131.03, 130.88, 129.01, 128.98, 128.90, 127.62, 127.28, 118.90, 112.79, 112.57, 106.27, 105.70, 105.55, 105.29, 105.03, 62.41, 11.73. IR (KBr, cm^−1^): 2923, 1642, 1613, 1560, 1497, 1268, 1091. ESI-HRMS (*m*/*z*) found: 480.0923 (calcd. for C_25_H_17_ClF_2_N_3_O_3_ [M + H]^+^: 480.0927).

*Compound* **4l**: Yellow solid; yield 78%; m.p.: 167–170 °C. ^1^H-NMR (600 MHz, DMSO-*d*_6_) δ 9.03 (s, 1H), 7.90 (d, *J* = 8.4 Hz, 2H), 7.81 (d, *J* = 8.4 Hz, 2H), 7.75 (q, *J* = 7.8 Hz, 1H), 7.49 (t, *J* = 8.3 Hz, 1H), 7.45 (t, *J* = 9.5 Hz, 1H), 7.31–7.23 (m, 1H), 7.20 (d, *J* = 8.4 Hz, 1H), 7.10 (d, *J* = 8.1 Hz, 1H), 5.42 (s, 2H), 2.64 (s, 3H). ^13^C-NMR (101 MHz, DMSO) δ 181.99, 155.64, 155.50, 146.74, 144.20, 136.18, 133.27, 130.87, 127.63, 123.29, 122.55, 121.93, 118.85, 112.79, 112.56, 106.12, 105.70, 105.55, 105.28, 105.02, 62.28, 11.81. IR (KBr, cm^−1^): 2925, 1648, 1612, 1565, 1498, 1270, 1254, 1085. ESI-HRMS (*m*/*z*) found: 546.0235 (calcd. for C_25_H_16_BrF_2_N_3_O_3_Na [M + Na]^+^: 546.0235).

*Compound* **4m**: Yellow solid; yield 80%; m.p.: 166–169 °C. ^1^H-NMR (600 MHz, DMSO-*d*_6_) δ 8.74 (s, 1H), 7.92 (d, *J* = 8.0 Hz, 1H), 7.79–7.73 (m, 1H), 7.68 (dd, *J* = 7.8, 1.6 Hz, 1H), 7.62 (t, *J* = 7.6 Hz, 1H), 7.56 (t, *J* = 7.7 Hz, 1H), 7.50 (t, *J* = 8.2 Hz, 1H), 7.45 (td, *J* = 10.0, 2.4 Hz, 1H), 7.27 (td, *J* = 8.5, 2.4 Hz, 1H), 7.20 (d, *J* = 8.4 Hz, 1H), 7.12 (d, *J* = 8.1 Hz, 1H), 5.45 (s, 2H), 2.64 (s, 3H). ^13^C-NMR (101 MHz, DMSO) δ 181.99, 162.75, 155.57, 146.75, 142.98, 136.58, 134.11, 132.52, 130.89, 129.46, 129.20, 127.65, 127.26, 119.35, 118.90, 112.78, 112.57, 106.27, 105.69, 105.55, 105.29, 105.02, 62.44, 11.76. IR (KBr, cm^−1^): 2924, 1645, 1613, 1597, 1560, 1266, 1253, 1086. ESI-HRMS (*m*/*z*) found: 546.0238 (calcd. for C_25_H_16_BrF_2_N_3_O_3_Na [M + Na]^+^: 546.0235).

*Compound* **4n**: Yellow solid; yield 78%; m.p.: 164–166 °C. ^1^H-NMR (600 MHz, DMSO-*d*_6_) δ 8.95 (s, 1H), 7.79 (d, *J* = 8.4 Hz, 2H), 7.75 (m, *J* = 8.3 Hz, 1H), 7.49 (t, *J* = 8.2 Hz, 1H), 7.45 (td, *J* = 10.4, 2.3 Hz, 1H), 7.39 (d, *J* = 8.1 Hz, 2H), 7.27 (td, *J* = 8.5, 2.5 Hz, 1H), 7.19 (d, *J* = 8.4 Hz, 1H), 7.10 (d, *J* = 8.1 Hz, 1H), 5.41 (s, 2H), 2.64 (s, 3H), 2.37 (s, 3H). ^13^C-NMR (101 MHz, DMSO) δ 181.98, 155.64, 155.54, 146.73, 143.88, 138.90, 134.74, 132.55, 130.87, 130.71, 127.64, 123.12, 120.48, 118.85, 112.76, 112.55, 106.10, 105.65, 105.28, 105.02, 62.34, 21.04, 11.80. IR (KBr, cm^−1^): 2325, 1649, 1612, 1564, 1498, 1271, 1256, 1086. ESI-HRMS (*m*/*z*) found: 460.1466 (calcd. for C_26_H_20_F_2_N_3_O_3_ [M + H]^+^: 460.1473).

*Compound* **4o**: Yellow solid; yield 75%; m.p.: 165–168 °C. ^1^H-NMR (600 MHz, DMSO-*d*_6_) δ 8.69 (s, 1H), 7.94 (s, 1H), 7.76 (td, *J* = 8.3, 6.5 Hz, 1H), 7.51–7.40 (m, 5H) 7.27 (td, *J* = 8.5, 2.5 Hz, 1H), 7.20 (d, *J* = 8.4 Hz, 1H), 7.11 (d, *J* = 8.1 Hz, 1H), 5.43 (s, 2H), 2.64 (s, 3H), 2.13 (s, 3H). ^13^C-NMR (101 MHz, DMSO) δ 181.99, 162.75, 155.64, 155.59, 146.75, 143.04, 136.65, 133.54, 132.67, 131.83, 130.88, 130.37, 127.63, 127.50, 126.66, 126.52, 118.89, 112.77, 112.57, 106.22, 105.66, 105.29, 62.54, 17.77, 11.71. IR (KBr, cm^−1^): 2924, 1641, 1610, 1563, 1500, 1269, 1090, 1039. ESI-HRMS (*m*/*z*) found: 460.1468 (calcd. for C_26_H_20_F_2_N_3_O_3_ [M + H]^+^: 460.1473).

*Compound* **4p**: Yellow solid; yield 85%; m.p.: 168–170 °C. ^1^H-NMR (600 MHz, DMSO-*d*_6_) δ 8.67 (s, 1H), 7.75 (td, *J* = 8.3, 6.5 Hz, 1H), 7.54 (td, *J* = 5.4, 5.0, 2.3 Hz, 1H), 7.51–7.48 (m, 2H), 7.47–7.43 (m, 1H), 7.42 (dd, *J* = 3.8, 1.2 Hz, 2H), 7.27 (td, *J* = 8.5, 2.5 Hz, 1H), 7.20 (d, *J* = 8.4 Hz, 1H), 7.11 (d, *J* = 8.1 Hz, 1H), 5.43 (s, 2H), 2.63 (s, 3H), 2.40 (q, *J* = 7.6 Hz, 2H), 0.95 (t, *J* = 7.6 Hz, 3H). ^13^C-NMR (101 MHz, DMSO) δ 182.00, 155.63, 155.56, 146.74, 143.06, 139.80, 136.12, 130.87, 130.74, 130.37, 127.61, 127.48, 126.98, 126.94, 118.91, 112.76, 112.55, 106.28, 105.66, 105.53, 105.28, 105.02, 62.53, 24.26, 15.16, 11.66. IR (KBr, cm^−1^): 2930, 1644, 1611, 1562, 1499, 1270, 1090. ESI-HRMS (*m*/*z*) found: 496.1453 (calcd. for C_27_H_21_F_2_N_3_O_3_Na [M + H]^+^: 496.1443).

*Compound* **4q**: Yellow solid; yield 79%; m.p.: 165–168 °C. ^1^H-NMR (600 MHz, DMSO-*d*_6_) δ 8.58 (s, 1H), 7.79–7.72 (m, 1H), 7.49 (t, *J* = 8.2 Hz, 1H), 7.47–7.43 (m, 1H), 7.39 (t, *J* = 7.6 Hz, 1H), 7.30–7.25 (m, 3H), 7.20 (d, *J* = 8.4 Hz, 1H), 7.09 (d, *J* = 8.0 Hz, 1H), 5.44 (s, 2H), 2.61 (s, 3H), 1.90 (s, 6H). ^13^C-NMR (101 MHz, DMSO) δ 181.99, 159.32, 155.59, 146.76, 143.09, 136.21, 135.30, 130.85, 130.48, 128.87, 127.57, 126.97, 118.93, 112.78, 112.58, 106.34, 105.66, 105.54, 105.28, 105.03, 62.70, 17.24, 11.55. IR (KBr, cm^−1^): 2924, 1643, 1610, 1559, 1498, 1268, 1090. ESI-HRMS (*m*/*z*) found: 474.1613 (calcd. for C_27_H_22_F_2_N_3_O_3_ [M + H]^+^: 474.1629).

*Compound* **4r**: Yellow solid; yield 79%; m.p.: 169–171 °C. ^1^H-NMR (600 MHz, DMSO-*d*_6_) δ 8.62 (s, 1H), 7.75 (q, *J* = 8.2 Hz, 1H), 7.49 (t, *J* = 8.2 Hz, 1H), 7.47–7.42 (m, 1H), 7.32 (d, *J* = 8.0 Hz, 1H), 7.28 (s, 2H), 7.20 (m, 2H), 7.11 (d, *J* = 8.1 Hz, 1H), 5.42 (s, 2H), 2.63 (s, 3H), 2.35 (s, 3H), 2.08 (s, 3H). ^13^C-NMR (101 MHz, DMSO) δ 181.99, 155.62, 146.74, 142.95, 140.02, 134.28, 133.18, 132.55, 132.23, 130.88, 127.89, 127.63, 126.67, 126.30, 118.89, 112.78, 112.56, 106.22, 105.65, 105.54, 105.28, 105.02, 62.54, 21.10, 17.67, 11.70. IR (KBr, cm^−1^): 2925, 1643, 1611, 1563, 1499, 1269, 1090. ESI-HRMS (*m*/*z*) found: 474.1623 (calcd. for C_27_H_22_F_2_N_3_O_3_ [M + H]^+^: 474.1629).

### 3.2. Antifungal Assay

Test fungal strains were obtained from China Pharmaceutical Culture Collection (*Candida albicans* CPCC400616), China General Microbiological Culture Collection Center (*Cryptococcus neoformans* CGMCC2.3161 and *Candida zeylanoides* CGMCC2.3739) or were clinical isolates (*Trichophyton rubrum* and *Rhodotorula rubra*).

The *in vitro* antifungal activity of target compounds was determined by the micro-broth dilution method in 96-well plates according to the CLSI M27-A3 guidelines [35]. Tested compounds and the control drug were dissolved in DMSO respectively and Tween-20 was added as a stabilizer. The prepared samples were serially two-fold diluted in growth medium, inoculated and incubated at 35 °C. The MICs were determined at 24 h for *C. alb.*, *C. zey*., *T. rub*., and *R. rub*., and at 72 h for *C. neo.*

## 4. Conclusions

In summary, a series of benzofuran-triazoles hybrids has been designed and synthesized. The target compounds were characterized by HRMS, FTIR and NMR. The biological assay results indicated that most of the target compounds possessed *in vitro* antifungal activity against fluconazole-resistant *Trichophyton rubrum* and *Cryptococcus neoformans*. Several compounds (e.g., **4o** and **4r**) showed satisfactory activity, which makes them valuable for further research. Based on these results, preliminary SARs were summarized to serve as a foundation for the further investigation of antifungal drugs.

## Figures and Tables

**Table 1:** Antifungal *in vitro* activities of the target compounds (MIC, μg·mL^−1^). 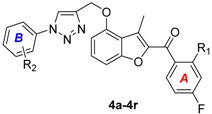

Compound	R_1_	R_2_	C. alb.	C. neo.	C. zey.	T. rub.	R. rub.
**2a**	H		>128	>128	>128	>128	>128
**2b**	F		>128	>128	>128	>128	>128
**4a**	H	4-Cl	>128	>128	>128	>128	>128
**4b**	H	2-Cl	>128	128	>128	128	>128
**4c**	H	4-Br	>128	>128	>128	>128	>128
**4d**	H	2-Br	>128	128	128	64	128
**4e**	H	4-CH_3_	>128	128	>128	64	>128
**4f**	H	2-CH_3_	128	64	64	64	128
**4g**	H	2-C_2_H_5_	>128	128	>128	128	>128
**4h**	H	2,6-di-CH_3_	128	128	64	64	128
**4i**	H	2,5-di-CH_3_	>128	128	>128	128	>128
**4j**	F	4-Cl	>128	128	>128	128	>128
**4k**	F	2-Cl	>128	64	>128	128	>128
**4l**	F	4-Br	>128	128	>128	128	>128
**4m**	F	2-Br	128	64	64	64	>128
**4n**	F	4-CH_3_	>128	64	>128	64	>128
**4o**	F	2-CH_3_	128	32	128	32	128
**4p**	F	2-C_2_H_5_	>128	64	64	64	>128
**4q**	F	2,6-di-CH_3_	>128	128	128	64	128
**4r**	F	2,5-di-CH_3_	128	64	64	32	128
FCZ			1	8	0.5	128	8

Abbreviations: C. alb., *Candida albicans* (CPCC400616); C. neo., *Cryptococcus neoformans* (CGMCC2.3161); C. zey., *Candida zeylanoides* (CGMCC2.3739); T. rub., *Trichophyton rubrum*; R. rub., *Rhodotorula rubra*; FCZ, Fluconazole.
